# Validating the Swedish STOP-D: a brief tool for depression, anxiety, stress, anger and social support

**DOI:** 10.3389/fpsyg.2025.1649601

**Published:** 2026-01-06

**Authors:** Andreas Larsson, Veronica Milos Nymberg, Peter Nymberg, Felicia T. A. Sundström

**Affiliations:** 1 Department of Psychology and Social Work, Mid Sweden University, Östersund, Sweden; 2 Center for Primary Health Care Research, Department of Clinical Sciences Malmö, Lund University, Malmö, Sweden; 3 University Clinic Primary Care, Skåne University Hospital, Region Skåne, Sweden; 4 Department of Psychology, Uppsala University, Uppsala, Sweden

**Keywords:** validating Swedish translation, screening mental health, depression single item, anxiety single item, stress single item, Ange single item, social support

## Abstract

Psychological distress, including depression, anxiety, and stress, requires time-efficient assessment tools suited for digital and momentary settings. The Screening Tool for Psychological Distress (STOP-D) is a five-item scale developed for brief mental health screening, but it has not yet been validated in Swedish. This study aimed to evaluate the psychometric properties and diagnostic accuracy of the Swedish STOP-D in a non-clinical adult sample. A total of 427 Swedish-speaking adults (mean age = 49.42, SD = 16.34 years) completed the STOP-D, the Patient Health Questionnaire-4 (PHQ-4), the Single-Item Stress Scale (SISE), and the Need Satisfaction and Frustration Scale (NSFS). ROC analyses were conducted using PHQ-2 and GAD-2 (cut-off ≥ 3) and the SISE (cut-off ≥ 6 and 7). Feature importance was examined using the Boruta algorithm. Exploratory Graph Analysis (EGA) assessed the internal structure. The STOP-D items for Sadness and Anxiety showed excellent classification accuracy for depression and anxiety (AUC = 0.87–0.89). Stress showed lower accuracy (AUC = 0.65–0.67). EGA supported a two-factor model, with STOP-D items for Sad, Anxiety and Stress clustering separately from Anger and Lack of Social Support. The Swedish STOP-D is a valid brief tool for assessing core psychological distress. Items for anger and social support may add clinical value and represent distinct processes relevant for future individualized and ecological assessment.

## Introduction

Psychological distress, encompassing a range of emotional states such as depression, anxiety, and stress, is a prevalent concern with substantial impacts on individuals and communities globally in both clinical and non-clinical contexts (e.g., Kessler and Ustun, 2008). Accurate assessment of psychological distress is pivotal for identifying, diagnosing, and addressing mental health issues in order to prevent but also treat them. In the evolving landscape of clinical psychology, assessing psychological distress in real-time contexts has become increasingly critical. Traditional measures like the Beck Depression Inventory-II (BDI-II; Beck et al., 2011) and the Montgomery-Åsberg Depression Rating Scale (MADRS; Montgomery and Asberg, 1979) offer depth but are often too extensive for real-time, dynamic assessments such as Ecological Momentary Assessment (EMA; e.g. Shiffman et al., 2008). EMA allows for the assessment of individuals in their natural environments, capturing the ebb and flow of psychological states in real-time.

The Screening Tool for Psychological Distress (STOP-D; Young et al., 2007) item set appears as a promising alternative, particularly when considering the need for brief measures in an EMA—style data collection. The STOP-D, contains five single-item scales for Sadness, Stress, Anger, Anxiety, and Social Support in a set, meaning that the items can be administered individually to suit the question in a clinical research setting.

Psychometric validation of item sets poses challenges because common approaches, such as assessing internal consistency, e.g., with Cronbach’s alpha or conducting Confirmatory Factor Analysis, require multiple items measuring the same underlying construct. In this context, each item operates independently as its own measure, making these traditional methods less appropriate.

To address this, alternative validation strategies are necessary. In the original validation of the STOP-D (Young et al., 2007) ROC curve analyses were used. Recent studies (Ciarrochi et al., 2022; Larsson and Sundström, 2024) have used machine learning techniques for feature selection, such as the Boruta package (Kursa and Rudnicki, 2010) in R, which allows for the evaluation of each item’s significance in predicting the outcome of interest.

The aim of this study is to translate and validate the STOP-D item set in a Swedish-speaking population. This will not only contribute to the broader endeavour of developing concise, time-efficient, culturally sensitive, and multidimensional tools for assessing psychological distress but also align with the growing trend of utilizing EMA in psychological research. By benchmarking against established measures like the PHQ-4, Single-Item Stress Measure, and the Need Satisfaction and Frustration Scale (NSFS), this study seeks to evaluate the Swedish-speaking STOP-D item set’s reliability, validity, and applicability. This endeavour aligns with the increasing demands for time-efficient and effective mental health care, enriching the repertoire of tools available for psychological assessment in Sweden and potentially in other similar cultural contexts.

## Materials and methods

### Participants

Participants were recruited via Qualtrics’s participant recruitment service and social media in April and May 2023. Inclusion criteria for participation were being over 18 years of age and Swedish speaking. No other specific exclusion criteria were specified.

The final sample consisted of 427 participants with a mean age of 48.03 years (SD = 17.06). Out of the final sample, 231 participants identified as female (mean age = 49.42, SD = 16.34) while 195 identified as male (mean age = 46.24, SD = 17.69), One participant identified as neither male nor female.

### Measures

#### Screening tool for psychological distress (STOP-D)

The STOP-D (Young et al., 2007) is a self-reported item tool designed to provide severity scores on five key areas of psychological distress: depression or sadness, anxiety, stress, anger, and poor social support. The translation followed a multi-step consensus process. The measure is self-administered and takes between 1 and 2 min to complete, requiring no scoring, and is free to use. The STOP-D has demonstrated robust psychometric properties in previous validation studies (Young et al., 2007, 2012). Analyses revealed high correlations between all STOP-D items and corresponding measures, with robust receiver operating characteristic curves. Specifically, severity scores on STOP-D-depression and STOP-D-anxiety correlated well with established severity cutoff scores on the Beck Depression Inventory and the Beck Anxiety Inventory, respectively. The STOP-D has proven to perform well when compared to other longer and validated measures, establishing its validity and reliability for assessing psychological distress in various populations (Young et al., 2007). The STOP-D was translated into Swedish using an established model (Beaton et al., 2000) of being translated by two experts in Psychology, Andreas Larsson (AL, PhD and Lic Psychologist), and Felicia Sundström, (FS, PhD Student and Lic Psychologist) and one non-expert, licensed teacher (Alice Furster). Minor variations were discussed, and consensus was easily reached. A back translation was performed by a professional translator (Lisa Cockette, Anything English), and reviewed for conceptual accuracy. A summary of the full translation process and back translation is available in the Supplemental material (Supplementary Data Sheet 1).

#### Patient health questionnaire-4 (PHQ-4)

The PHQ-4 is utilized as a screening instrument for both depression and anxiety disorders. It comprises two subscales: the PHQ-2 for depression and the GAD-2 for anxiety (REF). The measure has demonstrated reliability with a Cronbach’s alpha of α = 0.81 and its criterion validity has been established against other validated measures with a corrected item-total correlation ranging between *r* = 0.66 and *r* = 0.80, indicating good discriminative ability (Kroenke et al., 2009).

#### Single-item stress question (SISE)

Employed to gauge subjective stress over the past week, this measure has shown reliability with a test–retest correlation of *r* = 0.66 to 0.74 over studies, and its validity was comparable to longer questionnaires, making it a practical instrument for assessing stress in large prospective epidemiological studies (Arapovic-Johansson et al., 2017).

#### Need satisfaction and frustration scale (NSFS)

The NSFS is employed to evaluate three core psychological needs in line with Self-Determination Theory: Autonomy, Relatedness, and Competence. Psychometric validation of the NSFS has been substantiated in a study involving professional Romanian athletes (Alexe et al., 2022). Confirmatory factor analyses supported a six-factor correlated model, invariant across gender, age, and sport. The measure met criteria for convergent validity with average variance extracted values ranging between 0.60 and 0.74. Discriminant validity was evidenced by heterotrait–monotrait ratio of correlations among the six factors, with values ranging from −0.72 to 0.72. Reliability was endorsed by Cronbach’s alpha scores between 0.75 and 0.89, and Raykov’s composite reliability coefficient values between 0.76 and 0.89. Criterion validity was established through positive relationships of autonomy, competence, and relatedness satisfaction to autonomous motivation, and positive associations of autonomy, competence, and relatedness frustration with controlled motivation and amotivation.

### Data analysis

Given that the STOP-D constitutes an item set rather than a latent variable measure, the analysis primarily hinged on correlational analysis and feature selection. The correlational analysis was conducted using the Pearson correlation coefficient in JASP (version 0.18.0.0), examining the relationships between each item in the STOP-D item set and the criterion variables (Health, PHQ-4-Anxiety, PHQ-4-Depression, Single item stress, and the social support questions from the NSFS) individually. To control for Type I error due to multiple comparisons, a Bonferroni correction was applied.

For the three outcomes of PHQ-4-Depression, PHQ-4-Anxiety and SISE; ROC-analysis was utilized to assess specificity and sensitivity, and to suggest cut-offs for the Sad, Anxious and Stressed items, respectively, using the pROC (1.18.5) package. The cut-off points for the PHQ-4 subscales were set to ≤ 3 based on previous studies (Kroenke et al., 2009), and the SISE cut off was set to ≤ 7 (Arapovic-Johansson et al., 2017). The Anger and lack of social support items were not evaluated using ROC analysis due to the absence of established, validated external criteria in the current dataset. While these constructs are central to many psychological models, there is a lack of widely accepted short-form anchor measures with established clinical cutoffs in the Swedish context. Hence, the inclusion of these items in ROC-based validity testing was not feasible.

In order to examine the potential for unidimensionality of the five STOP-D items, Exploratory Graph Analysis (EGA) was conducted. EGA is a network psychometric method that estimates the number and structure of latent dimensions using a graphical lasso and community detection algorithm (Golino and Epskamp, 2017).

A machine learning feature selection package called Boruta (Kursa and Rudnicki, 2010) was utilized to assess the importance of each item in the STOP-D item set concerning the criterion variables. The Boruta algorithm generates shadow variables via Monte Carlo simulations from the actual data, thereafter running the combined dataset through a random forest plot to evaluate feature importance. The choice of Boruta is grounded in its demonstrated effectiveness in psychological research, where it has been used to identify key characteristics influencing patient outcomes in psychotherapy (Rubel et al., 2020). Furthermore, the versatility of this algorithm has been showcased in health-related fields, such as in the development of diabetes prediction models (Zhou et al., 2023), indicating its robustness in handling complex biomedical data. Separate feature selection analyses were conducted for each criterion variable, using the items from the STOP-D as features.

Handling of missing data was addressed using the Multivariate Imputation by Chained Equations (mice; Buuren and Groothuis-Oudshoorn, 2011) method, under the assumption that the data were Missing At Random (MAR). All analyses were conducted in R 4.4.3.

### Ethics

Ethical approval was a from the Swedish Ethical Review Authority (ref. 2022–07088-01). As the research was completely anonymized in Qualtrics, the Swedish Ethical Review Authority gave an advisory statement confirming that, according to the Swedish Ethical Review Act (SFS 2003:460), ethical vetting was not required for this type of study using anonymous data.

## Results

### Correlation analysis

The Pearson correlation coefficients among the STOP-D items, Health status, Anxiety, Depression, Stress, and NSFS variables were calculated. All correlations were significant at the *p* ≤ 0.001 level.

### STOP-D inter-item correlations

Table 1 presents the inter-item correlations among the STOP-D items. The coefficients ranged from 0.54 to 0.83, indicating a strong positive association among the STOP-D items in the expected directions.

**Table 1:** Mean and SD for STOP-D scores and inter-item Pearson’s correlation coefficients among STOP-D items.

Variable	Mean (SD)	Sad	Anxiety	Stress	Anger	Social support
Sad	42.67 (34.31)	—				
Anxiety	42.47 (34.44)	0.83***	—			
Stress	47.44 (34.01)	0.72***	0.80***	—		
Anger	36.52 (29.89)	0.67***	0.65***	0.66***	—	
Social support	40.97 (33.56)	0.62***	0.63***	0.57***	0.54***	—

*p ≤ 0.05; **p ≤ 0.001; ***p ≤ 0.0001.

### STOP-D and outcome correlations

Table 2 presents the correlations between the STOP-D items and the clinical outcome variables as well as the NSFS items, all correlations were statistically significant at the *p* ≤ 0.001 level. Correlations were moderate to strong between the STOP-D items and Health (*r* = −0.40 to −0.23), Anxiety (*r* = 0.63 to 0.71), Depression (*r* = 0.68 to.67) and Stress (*r* = 0.63 to.71). This suggests that higher levels of STOP-D items: Sadness, Anxiety, Stress, Anger and Lack of Social Support are associated with poorer health outcomes and higher anxiety, stress, and depression.

**Table 2:** Mean and SD for STOP-D scores and Pearson’s correlation coefficients (*r*) between STOP-D items, clinical outcome variables, and NSFS variables.

STOP-D	Mean (SD)	Sad	Anxiety	Stress	Anger	Social support
Clinical outcomes						
Health	2.56 (1.09)	−0.40***	−0.39***	−0.33***	−0.23***	−0.32***
GAD-2	2.15 (1.96)	0.63***	0.71***	0.65***	0.53***	0.56***
PHQ-2	2.23 (1.94)	0.68***	0.67***	0.58***	0.54***	0.57***
SISQ	3.00 (1.29)	0.63***	0.70***	0.71***	0.53***	0.56***
Needs satisfaction and frustration scales						
Autonomy frustration	2.60 (1.19)	0.45***	0.49***	0.52***	0.41***	0.42***
Autonomy Satisfaction	3.31 (1.14)	−0.39***	−0.37***	−0.43***	−0.32***	−0.26***
Competency frustration	2.74 (1.18)	0.50***	0.52***	0.49***	0.39***	0.47***
Competency Satisfaction	3.25 (1.07)	−0.34***	−0.37***	−0.32***	−0.25***	−0.27***
Social Frustration	2.68 (1.26)	0.52***	0.54***	0.48***	0.40***	0.49***
Social Satisfaction	3.37 (1.11)	−0.32***	−0.28***	−0.25***	−0.17***	−0.35***

*p ≤ 0.05; **p ≤ 0.01; ***p ≤ 0.001. PHQ, Patient health questionnaire; SISQ, Single Item Stress Questionnaire; AF, Putonomy Frustration; AS, Autonomy Satisfaction; CF, Competency Frustration; CS, Competency Satisfaction; SF, Social Frustration; SS, Social Satisfaction.

The NSFS variables show a mixed pattern of relationships with emotional states. For example, Autonomy Frustration (*r* = 0.45 to 0.52) and Competency Frustration (*r* = 0.50 to 0.52) exhibit moderate positive correlations with negative emotional states, suggesting that frustration in autonomy and competency is linked to higher levels of negative emotions. On the other hand, Autonomy Satisfaction and Competency Satisfaction show negative correlations (AS; *r* = −0.39 to −0.37; CS; *r* = −0.34 to −0.37), indicating that satisfaction in these domains may act as a buffer against negative emotions. Social Frustration correlates positively with negative emotional states (*r* = 0.52 to 0.54), whereas Social Satisfaction shows negative correlations (*r* = −0.32 to −0.28), emphasizing the significance of social factors in psychological well-being.

### Specificity, sensitivity and dimensionality

Using the PHQ-2 depression subscale (cutoff ≥3), the Sad item showed strong performance with an AUC of 0.87 (95% CI: 0.83–0.91), optimal cutoff of 18.0, sensitivity = 0.83, and specificity = 0.82 (Youden = 1.63). Likewise, the Anxiety item, using the GAD-2 anxiety subscale (cutoff ≥3), also showed strong accuracy with an AUC of 0.89 (95% CI: 0.85–0.93), optimal cutoff of 12.0, sensitivity = 0.86, and specificity = 0.82 (Youden = 1.70).

The stress item displayed more modest predictive capacity at a SISE cut-off of ≥7; the AUC was 0.65 (95% CI: 0.54–0.76), with a sensitivity and specificity of approximately 0.67. With a lowered SISE threshold (≥6), AUC slightly improved to 0.67 and specificity increased to 0.72, though sensitivity decreased to 0.67. A suggested cut-off would be 26.5 (Youden = 1.33).

**Figure 1:**
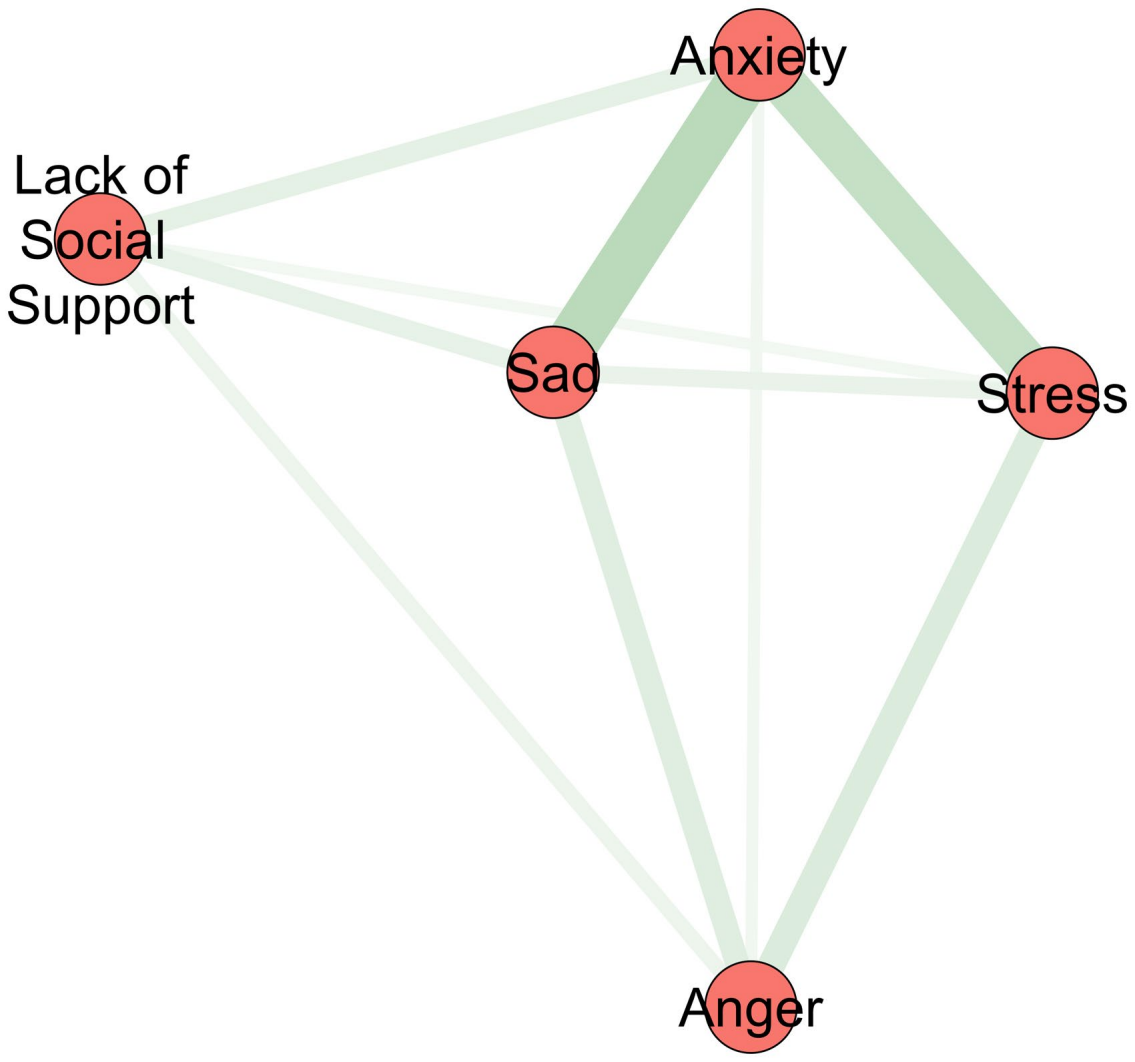
EGA network of STOP-D items.

EGA indicated that the Anger and Lack of Social Support items clustered separately from the core distress indicators (Sad, Anxiety and Stress; Figure 1), suggesting distinct latent dimensions corresponding to emotional regulation and interpersonal support.

### STOP-D item importance

The importance of each STOP-D item in predicting the various outcomes was evaluated using the Boruta feature selection method. The rankings are presented in Table 3, where a rank of 1 indicates the highest importance and a rank of 5 indicates the lowest importance. The “-” notation denotes that the STOP-D item was not identified as an important feature for the corresponding outcome. The results demonstrate that all STOP-D items are important features for the outcomes Health, Vitality, Depression, Anxiety and Social Frustration, but that the Stress and Anger items did not outperform the shadow variables on Social Satisfaction.

**Table 3:** Ranked importance for the Stop-D items for relevant outcomes.

STOP-D item	Health	Vitality	PHQ-4 Dep	PHQ-4 anxiety	Social frustration	Social satisfaction
1. Sad	2	1	1	1	1	3
2. Anxiety	3	2	2	2	2	4
3. Stress	4	3	3	3	3	-
4. Anger	1	4	4	4	4	-
5. Social Support	5	5	5	5	5	5

PHQ, Patient Health Questionnaire. 1 is most important, and “NO” indicates that the variable did not outperform shadow variables.

## Discussion

The present study validated the Swedish version of the Screening Tool for Psychological Distress (STOP-D) in a community sample of Swedish-speaking adults. The results provide strong support for the use of the STOP-D as a brief screening tool, particularly for symptoms of depression and anxiety.

Sadness and Anxiety demonstrated excellent classification accuracy when benchmarked against the PHQ-2 and GAD-2 subscales, with AUC values of 0.87 and 0.89, respectively. These results suggest that the STOP-D can accurately identify individuals with elevated symptoms of depression and anxiety using only two single items. Stress, while still significant, showed moderate classification accuracy against the Single Item Stress Scale, which may reflect either the less specific nature of the criterion or the contextual variability of stress.

Exploratory Graph Analysis revealed that the items Sadness, Anxiety, and Stress grouped together in one cluster, while Anger and Lack of Social Support formed a distinct second cluster. This suggests that the latter two items reflect processes that may function differently from internalizing symptoms, such as emotion regulation and social connection. This pattern aligns with prior research suggesting a distinction between internalizing symptomatology and broader process-based constructs such as affective regulation and interpersonal functioning. In the context of process-based therapy, the first cluster may reflect momentary subjective states, while the second points to contextual processes that shape how distress is maintained or buffered over time.

The Boruta feature selection algorithm confirmed the importance of all five items across multiple outcomes, though Anger and Lack of Social Support were somewhat less predictive for criteria like social satisfaction. This finding underscores the potential utility of these items beyond traditional mental health screening, particularly in understanding the broader psychosocial context of distress. For instance, although both Anger and Lack of Social Support were retained as important features by the Boruta algorithm, their lower relative importance and non-confirmation for social satisfaction suggest that they may be less robust predictors of traditional mental health outcomes, while still offering clinically relevant process information.

There are several limitations to note. First, the cross-sectional design does not allow for conclusions about sensitivity to change or test–retest reliability. Second, Anger and Lack of Social Support could not be evaluated using ROC analyses due to the lack of validated external anchors for anger and social support. Third, the sample was recruited online and was non-clinical, which may limit generalizability to clinical or more diverse populations.

From a practical perspective, the STOP-D shows promise for rapid screening in primary care, workplace health assessments, and digital mental health platforms. Its brevity makes it suitable for ecological momentary assessment (EMA) and repeated measurement, and its multidimensional nature allows users to gain insight into emotional, behavioral, and social processes in real time.

Future research should assess the responsiveness of STOP-D items over time, particularly in intervention studies using ecological momentary assessment. Establishing normative cutoffs and validating Anger and Lack of Social Support against external measures such as the STAXI-2 and MSPSS would further strengthen the tool. Additional analyses using bifactor models or bridge centrality may also help clarify the unique contribution of each item to overall distress profiles.

While the guidelines by the American Educational Research Association, American Psychological Association, and National Council on Measurement in Education (2011) recommend thorough content-based validity procedures for psychological instruments, such methods are not always appropriate for ultra-brief, single-item scales like the STOP-D. For such tools, it may be more appropriate to assess conceptual clarity through expert consensus and translation procedures, alongside criterion validity. Our strategy followed the original validation approach, emphasizing brief, translatable content and empirical associations with relevant outcomes.

In conclusion, the Swedish STOP-D demonstrates strong psychometric properties as a brief screening instrument for psychological distress, especially in contexts requiring frequent or low-burden assessment, such as app-based interventions, occupational health check-ins, or stepped care systems, especially for depression and anxiety. The inclusion of items related to anger and social support may offer additional clinical insights and warrants further investigation in more diverse and longitudinal contexts.

## Data Availability

The original contributions presented in the study are included in the article/Supplementary material, further inquiries can be directed to the corresponding author.
