# Supplementary material for: Validating the Swedish STOP-D: a brief tool for depression, anxiety, stress, anger and social support
Source: Front Psychol. 2026 Jan 6;16:1649601. doi: 10.3389/fpsyg.2025.1649601 (PMC12815834; doi:10.3389/fpsyg.2025.1649601)
Supplement: Supplementary file 1 [file Data_Sheet_1.PDF]

Translation process of the STOP-D from English to Swedish

Note from consensus calibration discussion: The five items were translated separately by two Swedish native speakers who are also licensed psychologists, one is a university lecturer and PhD, the other a PhD student. They are from different universities. A licensed teacher who also is a native Swedish speaker also made the translation. The results were the same wording for the first four items and difference in the last one were minimal, the more conservative option of keeping the more abrupt workding of "not having" which in Swedish required the addition of "att du" to make the flow easier. This was quickly agreed upon.

Orignal wording	*Swedish consensus wording*	*Back translation*
1. Feeling sad, down, or uninterested in life	Känt dig ledsen, nedstämd eller ointresserad av livet	Felt sad, down, or apathetic to life
2. Feeling anxious or nervous	Känt dig ångestfylld eller nervös	Feeling anxious or nervous
3. Feeling stressed	Känt dig stressad	Feeling stressed
4. Feeling angry	Känt dig arg	Feeling angry
5. Not having the social support you feel you need	Att du inte har haft det sociala stöd som du känner att du behöver	That you have not had the social support that you feel you need

Note: The Swedish version retains the meaning but uses natural idiomatic phrasing suitable for public-health or clinical contexts. The back translation confirms conceptual equivalence, showing only minor lexical variation ("apathetic" vs. "uninterested"), which is acceptable.
