# Supplementary material for: Validating the Swedish STOP-D: a brief tool for depression, anxiety, stress, anger and social support
Source: Front Psychol. 2026 Jan 6;16:1649601. doi: 10.3389/fpsyg.2025.1649601 (PMC12815834; doi:10.3389/fpsyg.2025.1649601)
Supplement: Supplementary file 2 [file Data_Sheet_2.PDF]

The Screening Tool for Psychological Distress (STOP-D) Svensk version

Under den senaste veckan. hur mycket har du besvärats av följande?

Instämmer helt	Instämmer inte alls										
	0	10	20	30	40	50	60	70	80	90	100
Känt dig ledsen. nedstämd eller ointresserad av livet											
Känt dig ångestfylld eller nervös											
Känt dig stressad											
Känt dig arg											
Att du inte har haft det sociala stöd som du känner att du behöver											

Översatt 2023 av Andreas Larsson, Felicia Sundström och Alice Furster.
